# Urinothorax after ultrasonography-guided renal biopsy: a case report

**DOI:** 10.1186/s12882-018-0903-8

**Published:** 2018-05-03

**Authors:** Tae Won Lee, Ha Nee Jang, Hyun Seop Cho, See Min Choi, Bong-Hoi Choi, Eunjin Bae, Se-Ho Chang, Dong Jun Park

**Affiliations:** 1 Department of Internal Medicine, Changwon Gyeongsang National University Hospital, 11, Samjeongja-ro, Seongsan-gu, Changwon-si, Gyeongsangnam-do South Korea; 2 Department of Internal Medicine, Gyeongsang National University Hospital, Jinju, Republic of Korea; 3 Department of Urology, Gyeongsang National University Hospital, Jinju, Republic of Korea; 4 Department of Nuclear and Molecular imaging, Gyeongsang National University Hospital, Jinju, Republic of Korea; 5 Department of Internal Medicine, Gyeongsang National University School of Medicine, 90, Chiram-dong, Jinju-si, Gyeongsangnam-do South Korea; 6 Institute of Health Science, Gyeongsang National University, Jinju, Republic of Korea

**Keywords:** Urinothorax, Pleural effusion, Renal biopsy

## Abstract

**Background:**

Urinothorax is defined as the presence of urine in the pleural space and is a rather rare cause of transudate pleural effusion. The potential etiologies are urinary tract obstruction and trauma. Diagnosis requires a high index of clinical suspicion and the condition is completely reversible following relief of underlying disease.

**Case presentation:**

We report a 27-year-old man who developed urinothorax after renal biopsy. Urine leakage was confirmed with ^99m^Tc DTPA (diethylenetriaminepentacetate) and single-photon emission computed tomography scans and retrograde pyelography. The pleural effusion was completely resolved by removing the leakage with a Foley catheter and a double J stent.

**Conclusions:**

Urinothorax has not been reported in patients doing renal biopsy in the literature. Based on our experience, urinothorax should be suspected, diagnosed, and managed appropriately when pleural effusion occurred after renal biopsy.

**Electronic supplementary material:**

The online version of this article (10.1186/s12882-018-0903-8) contains supplementary material, which is available to authorized users.

## Background

Urinothorax refers to the presence of urine in the pleural space, but rarely causes pleural effusion [1]. Since the first description in 1968 by Corriere et al., fewer than 70 cases have been reported worldwide [2]. Urinothorax is usually divided into obstructive urinothorax, due to obstructive uropathy resulting from calculi; prostatic hypertrophy; genitourinary malignancy; and traumatic urinothorax, which occurs following blunt trauma, ureteral instrumentation, surgery, or extracorporeal shock wave lithotripsy [3]. It is the cause of transudative pleural effusion secondary to obstructive uropathy; the effusion resolves quickly after removing the urinary tract obstruction. Although definite diagnostic criteria for urinothorax have not been established, the biochemical characteristics of the pleural fluid are useful [3–5]. A low index of suspicion and a greater emphasis on severe clinical conditions, such as urologic complications, can make it difficult to diagnose early, resulting in severe clinical situations, such as a large volume drainage after inserting a chest tube and respiratory failure. Here, we discuss a case of urinothorax occurring after ultrasonography (USG)-guided renal biopsy, which was diagnosed late due to unawareness of this relationship.

## Case presentation

A 27-year-old Korean male was admitted to the emergency room due to progressive shortness of breath and fever on the day of a hospital visit. The patient had been conducted as follows. The patient was placed in the prone position. The biopsy was taken from the lower pole of the kidney below 12th rib. The biopsy needle (16 gauge) is guided using USG to ensure visualization of the needle as it pierces the kidney parenchyme. There had been no immediate complications. Biopsy result revealed idiopathic membranous nephropathy and he received conservative management, including a low salt and protein diet, 80 mg of furosemide, and 80 mg of valsartan for 3 months. He had also been admitted to the department of nephrology due to transudative left pleural effusion, which was considered a complication of nephrotic syndrome and was discharged with full expansion of the lung by percutaneous drainage 3 weeks prior. He had no history of tuberculosis, chronic hepatitis, hypertension, or diabetes.

A physical examination revealed an alert status, but an acute-ill looking appearance. A marked decrease in breathing sounds and dullness upon percussion of the left side of the chest without wheezing was detected. His heart beat was regular without a murmur. Respiratory rate was 22/min, blood pressure was 130/80 mmHg, pulse rate was 115/min, and body temperature was 38.3 °C. Oxygen saturation was 95% on room air. No palpable lymph nodes were detected in the head or neck area. Organomegaly was not seen in the abdomen. No pretibial pitting edema, muscular swelling, or skin color changes were observed on either lower extremity. No tenderness on either costovertebral angle area was noted.

**Fig. 1:**
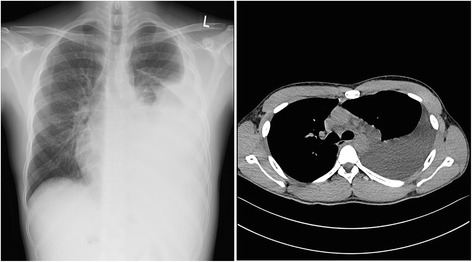
Chest PA and chest CT showing left-sided pleural effusion

A chest X-ray and computed tomography (CT) scan revealed massive left-sided pleural effusion (Fig. 1). A complete blood count revealed hemoglobin of 13.8 g/dL; total leukocyte count, 17.7 × 10^3^/mm^3^ with predominant neutrophils (77%); and a platelet count of 238 × 10^3^/mm^3^. The blood biochemistry profile was as follows: protein, 4.4 g/dL; albumin, 2.1 g/dL, cholesterol, 223 mg/dL; glucose, 92 mg/dL; lactic dehydrogenase (LDH), 190 U/L; alkaline phosphatase, 46 IU/L; aspartate aminotransferase, 16 IU/L; alanine aminotransferase, 20 IU/L; blood urea nitrogen, 18.1 mg/dL; and creatinine, 0.85 mg/dL. His C-reactive protein level was 3.9 mg/L. A microurinalysis revealed 3+ protein and 2+ blood on a dipstick and 10–19 red blood cells/high-power field. The spot urine protein/creatinine ratio was 4.7.

A 14 Fr pigtail catheter was inserted into the pleural cavity, and 2 L of pleural fluid was drained initially over 24 h. A biochemical analysis of the pleural fluid revealed pH, 7.8; glucose, 93 mg/dL; LDH, 60 U/L; creatinine, 1.1 mg/dL; protein, 0.2 g/dL; amylase, 17 U/L; and adenosine deaminase, 2 IU/L. The pleural fluid/serum creatinine ratio was 1.29. Cultures from blood, pleural fluid, and urine were sterile. Cytology from the pleural fluid did not show any malignant cells. The patient was started on intravenous empirical antibiotics. His clinical manifestations, such as respiratory stress and fever, nearly disappeared on day 3 after admission.

**Fig. 2:**
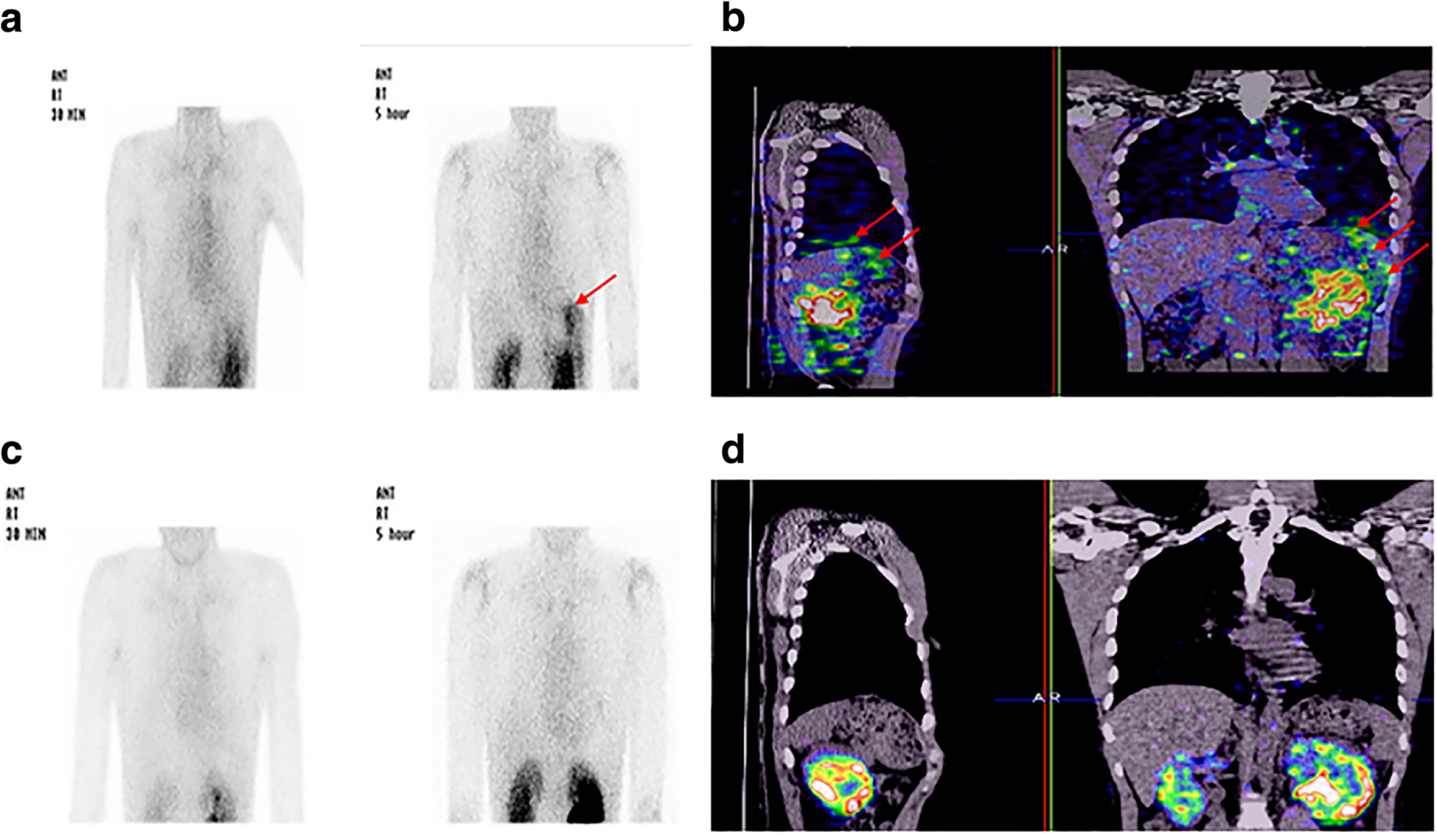
**a** Initial ^99m^Tc DTPA: Not 30 min, but 5 h posterior static image showing significant buildup of trace in the left sub-diaphragm and pleural space (arrow). **b** Initial SPECT-CT: Not 30 min, but 5 h scanning image demonstrating tracer uptake in the right sub-diaphragm and pleural space (arrow). **c** Follow up ^99m^Tc DTPA: 5 h posterior static image showing complete disappearance of buildup of trace in the right sub-diaphragm and pleural space. **d** Follow up SPECT-CT: 5 h scanning image demonstrating disappearance of tracer uptake in the right sub-diaphragm and pleural space

An enhanced CT scan on day 6 after admission revealed diffuse left renal swelling and fluid collection in the anterior pararenal space. No specific urine leakage was seen on intravenous pyelography. There was no distal obstruction on these studies. Under suspicion of urinothorax, a ^99m^Tc DTPA (diethylenetriaminepentacetate) renal scan was performed on day 12 after admission. Radionuclide scintigraphy and single-photon emission computed tomography (SPECT-CT) using ^99m^Tc DTPA revealed urine leakage from the lower pole of the left kidney to the left sub-diaphragm and the pleural space 5 h after injecting the tracer (Fig. 2). This leakage was also confirmed through retrograde pyelography (RGP) (Additional file 1: Fig. S1). A double-J stent and Foley catheter were inserted for 4 weeks to reduce leakage pressure and he was tolerable with this intervention. Follow-up radionuclide scintigraphy and SPECT-CT did not show any kidney or tracer leakage at the sub-diaphragm or pleural space (Fig. 2). The pigtail catheter was removed with full lung expansion, and the double-J stent and Foley catheter were taken out safely. He is being followed in our outpatient department with no recurrence of urinothorax for 1 year.

## Discussion

Here, we report the first case of urinothorax occurring after USG-guided renal biopsy. The patient had been suffering from pleural effusion at the third admission after the second discharge for pleural effusion, because we initially did not suspect a relationship between the pleural effusion and the renal biopsy results. We finally confirmed urine leakage into the pleural space resulting from urinoma with the help of appropriate tests including ^99m^Tc DTPA and SPECT-CT scans, as well as RGP. The pleural effusion resolved completely after inserting a Foley catheter and double-J stent.

Urine can reach the pleural space from the retroperitoneal space via lymphatic drainage [3, 5, 6] or with direct movement of the abdominal fluid into the pleural space through anatomical defects in the diaphragm, which is the dominant mechanism for a hydrothorax in patients with cirrhosis and ascites [3, 5–7]. The rapid accumulation of pleural fluid, which is common in patients with urinothorax, demonstrates that the latter may have been the dominant mechanism in our patient. The radionuclide scintigraphy and SPECT-CT scan performed using ^99m^Tc DTPA showed that high amounts of tracer were linked between the kidney, the left sub-diaphragm, and the left thorax 5 h after injecting the tracer. This suggests that the urinothorax mechanism may be due to direct movement from the retroperitoneum to the pleural cavity via a diaphragmatic defect. SPECT-CT was a good diagnostic tool to show the tracer flow two-dimensionally. No previous study has demonstrated urinothorax using SPECT-CT.

Urinothorax is classically classified as obstructive or traumatic [3, 5]. Obstructive one is associated with a bilateral or a common distal obstructive disease. Reported cases include prostate disease, bladder cancer or metastatic involvement, urethral valves, and urethral perforation. Traumatic one can be divided into two categories depending on causes again; accidental or iatrogenic. Reported cases for traumatic urinothorax are as follows: surgical injury, blunt trauma, percutaneous nephrostomy, and renal calculi lithotripsy [3, 5]. Urinothorax after percutaneous nephrostomy, which is quite a similar procedure to our case although it is more invasive has been reported. Recently, two cases of urinothorax after percutaneous nephrostomy has been reported [8, 9]. One case occurred following a percutaneous nephrostomy in the presence of non-draining stent [8]. The other case was secondary to ureteric obstruction and a previous percutaneous nephrostomy [9]. Stent insertion and ureteric obstruction was not in our case.

The diagnosis of urinothorax initially starts with a high degree of clinical suspicion. If the clinical manifestations are suggestive, a pleural fluid biochemical analysis can be helpful. The biochemical characteristics of urinothorax are related to those of urine due to the movement of urine, its low cell content, wide range in pH depending on food consumed, high creatinine level, low glucose level, very low protein, and high LDH level [3–5]. Overall, pleural effusion from urinothorax is transudative. However, the fluid that passes into the pleural space differs from urine but is almost identical to serum as times goes on. Therefore, early thoracentesis and analysis cannot be overemphasized for an accurate diagnosis.

An elevated pleural fluid/serum creatinine ratio > 1 has been commonly considered a hallmark in urinothorax [4, 5–7, 10]. However, this criterion may not be specific to urinothorax. One report showed that 12% of patients without urinothorax had a ratio > 1 [3]. Tudik et al. and Gutierrez-Rubio et al. also demonstrated that about 40% and 19% patients had ratios > 1, respectively [11, 12]. In contrast, a higher serum creatinine level was observed in some urinothorax cases, compared with pleural creatinine concentration [10]. The wide range of pleural fluid/serum creatinine ratios may depend on the time interval from initiation of urinothorax to thoracentesis. Later values are lower because high pleural creatinine level can be diluted by increased pleural effusion. Therefore, very high values, which might be acquired by early intervention, seem to be very specific to urinothorax, although values close to 1 cannot completely exclude urinothorax. The lower ratio in our case may have been due to the later diagnosis, resulting in the large pleural effusion.

A very high LDH level has been found in several cases of urinothorax [3–6]. This finding is so frequent that it may be misclassified as an exudate, and the clinician may not consider or could rule out urinothorax. However, the increased LDH level may be associated with the underlying urinary process that produces the rise in activity of this enzyme. One report demonstrated that urinary LDH level is a non-specific marker of cellular disruption anywhere along the urinary tract [13]. Pleural fluid LDH level is not correlated with other biochemical parameters in patients with urinothorax [5]. The pleural LDH level was extremely low in our case because there was no underlying urinary process to elevate the LDH level.

Urinothorax treatment is relatively straightforward as it is directed toward correcting the underlying disease. If underlying disease are not correctable, prompt diversion or re-establishing distal drainage are the important principles of treatment. Draining the pleural effusion is necessary if the patient is symptomatic. Because urine leakage by kidney biopsy was the cause of urinothorax in our case, our management was focused on healing the leakage through the replacement of the Foley catheter and double-J stent, resulting in reduced leakage pressure. Periodic radionuclide scintigraphy was performed to check for urine leakage and the Foley catheter and double-J stent were removed after the urine leakage was resolved. Conservative management by replacing the Foley catheter and double-J stent may be sufficient to control urine leakage in the case of urinothorax from renal biopsy, as opposed to a more invasive technique.

## Conclusions

In conclusion, the diagnosis and treatment of urinothorax begins with a high degree of suspicion. The clinician should keep in mind that urinothorax may occur after renal biopsy. When urinothorax is suspected, an early thoracentesis is indicated to confirm the biochemical characteristics such as pH, glucose, protein, creatinine, and LDH. In addition, a simultaneous blood sample should be taken to measure the pleural fluid/serum creatinine ratio. Early diagnosis and proper management targeted at the underlying disease should be performed to evade severe complications.

## Additional file

Additional file 1: **Figure S1.** Retrograde pyelography (RGP) showing urine leakage from lower pole of kidney. (TIF 110 kb)

## Abbreviations

CT: Computed tomography
DTPA: Diethylenetriaminepentacetate
LDH: Lactic dehydrogenase
SPECT-CT: Single-photon emission computed tomography
RGP: Retrograde pyelography
USG: Ultrasonography
