# Supplementary figures and images for: Urinothorax after ultrasonography-guided renal biopsy: a case report
Source: BMC Nephrol. 2018 May 3;19:104. doi: 10.1186/s12882-018-0903-8 (PMC5934790; doi:10.1186/s12882-018-0903-8)

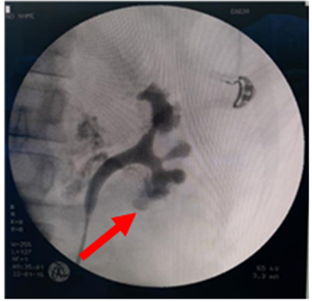

Supplement: Supplementary file 1 — Figure S1. Retrograde pyelography (RGP) showing urine leakage from lower pole of kidney. (TIF 110 kb) [file 12882_2018_903_MOESM1_ESM.tif]
